# The Risk of Type 2 Diabetes Mellitus in a Russian Population Cohort According to Data from the HAPIEE Project

**DOI:** 10.3390/jpm11020119

**Published:** 2021-02-11

**Authors:** Svetlana V. Mustafina, Oksana D. Rymar, Liliya V. Shcherbakova, Evgeniy G. Verevkin, Hynek Pikhart, Olga V. Sazonova, Yuliya I. Ragino, Galina I. Simonova, Martin Bobak, Sofia K. Malyutina, Mikhail I. Voevoda

**Affiliations:** 1 Institute of Internal and Preventive Medicine–Branch of Institute of Cytology and Genetics, Siberian Branch of Russian Academy of Sciences, 630089 Novosibirsk, Russia; orymar23@gmail.com (O.D.R.); 9584792@mail.ru (L.V.S.); ewer@mail.ru (E.G.V.); ragino@mail.ru (Y.I.R.); yu.p.nikitin@gmail.com (G.I.S.); smalyutina@hotmail.com (S.K.M.); mvoevoda@ya.ru (M.I.V.); 2 Institute of Epidemiology and Health Care, University College London, Gower Street, London WC1E6BT, UK; h.pikhart@ucl.ac.uk (H.P.); m.bobak@ucl.ac.uk (M.B.); 3 Novosibirsk State Medical University, 630091 Novosibirsk, Russia; ov_sazonova@mail.ru

**Keywords:** diabetes mellitus, risk factor, diabetes risk scale, diabetes risk model

## Abstract

The aim of this study is to investigate the 14-year risk of type 2 diabetes mellitus (T2DM) and develop a risk score for T2DM in the Siberian cohort. A random population sample (males/females, 45–69 years old) was examined at baseline in 2003–2005 (Health, Alcohol, and Psychosocial Factors in Eastern Europe (HAPIEE) project, *n* = 9360, Novosibirsk) and re-examined in 2006–2008 and 2015–2017. After excluding those with baseline T2DM, the final analysis included 7739 participants. The risk of incident T2DM during a 14-year follow-up was analysed using Cox regression. In age-adjusted models, male and female hazard ratios (HR) of incident T2DM were 5.02 (95% CI 3.62; 6.96) and 5.13 (95% CI 3.56; 7.37) for BMI ≥ 25 kg/m^2^; 4.38 (3.37; 5.69) and 4.70 (0.27; 6.75) for abdominal obesity (AO); 3.31 (2.65; 4.14) and 3.61 (3.06; 4.27) for fasting hyperglycaemia (FHG); 2.34 (1.58; 3.49) and 3.27 (2.50; 4.26) for high triglyceride (TG); 2.25 (1.74; 2.91) and 2.82 (2.27; 3.49) for hypertension (HT); and 1.57 (1.14; 2.16) and 1.69 (1.38; 2.07) for family history of diabetes mellitus (DM). In addition, secondary education, low physical activity (PA), and history of cardiovascular disease (CVD) were also significantly associated with T2DM in females. A simple T2DM risk calculator was generated based on non-laboratory parameters. A scale with the best quality included waist circumference >95 cm, HT history, and family history of T2DM (area under the curve (AUC) = 0.71). The proposed 10-year risk score of T2DM represents a simple, non-invasive, and reliable tool for identifying individuals at a high risk of future T2DM.

## 1. Introduction

In the last decades, the prevalence of diabetes mellitus (DM) has consistently risen in the general world population, thus making DM a medical and social problem worldwide [1]. According to the International Diabetes Federation forecast in 2019, the number of subjects with diabetes is expected to reach 578 million by 2030 and 700 million by 2045 [2]. In the Russian Federation, the prevalence of DM is also rising. According to the Federal Register of Diabetes Mellitus, in Russia by the end of 2016, 4.35 million subjects (3.0% of the population) had been registered in a dispensary as DM patients, of which 92% (4 million) had type 2 diabetes mellitus (T2DM), 6% (255,000) had type 1 diabetes mellitus, and 2% (75,000) other types of diabetes [3]. According to the NATION study, among the adult Russian population of 20–79 years old, 5.4% have T2DM. Moreover, approximately one-half of patients with diagnosed DM (54%) were unaware of this disease [4]. In our baseline survey within the Health, Alcohol, and Psychosocial Factors in Eastern Europe (HAPIEE) project, 2003–2005, we found a high prevalence of T2DM (11.3%) in the population sample aged 45–69 years old in Novosibirsk [5]. The rate is close to the data on compatible age groups in the NATION study conducted in Russia in 2013–2015 [4].

T2DM is known to be a multifactorial disease, and environmental factors are important for T2DM pathogenesis. The risk factors of T2DM are well established and include abdominal obesity (waist circumference (WC) ≥94 cm in males and ≥ 80 cm in females), a family history of DM, age >45 years old, hypertension and major cardiovascular diseases (CVDs), gestational diabetes, the use of drugs that contribute to hyperglycaemia, and weight gain. Early identification of T2DM risk factors and their clusters with the aim of modification can help to prevent T2DM [6,7]. At present, preventive strategies rely on the identification of risk factors and their combinations and subsequent lifestyle intervention. Appropriate lifestyle changes, including the normalisation of diet, increased physical activity, and weight loss can reduce the risk of T2DM by as much as 56% [8].

One of the first tools to identify individuals at high risk for T2DM is the Finnish diabetes risk score (FINDRISC) [9,10]. This tool was later successfully validated in other countries, including Germany, Holland, Denmark, Sweden, England, and Australia, [10,11]. The results show good sensitivity (Se) and specificity (Sp) in Germany, the USA, Switzerland, and Canada [12,13,14,15], although it did not perform well among the Omani Arabs [16].

To prevent further increase in the prevalence of diabetes, the identification of individuals at high risk for hyperglycaemia using inexpensive and available methods is crucial. Using risk score methods of prediction allows us to set the level of total risk, identify high-risk patients, and prescribe the necessary preventive measures.

Risk factors of T2DM have been studied in healthcare institutions and cross-sectional studies in Russia. Nonetheless, we are not aware of a Russian prospective cohort analysis of the long-term risk of T2DM in the general population. In this study, we aimed to investigate the 14-year risk of T2DM in a Russian population cohort in order to develop a risk scale to predict the development of T2DM over 10 years in people aged 45–69 years old.

## 2. Materials and Methods

### 2.1. Study Population and Methods

The data came from the Russian arm of the HAPIEE project [17]. The cohorts in this multicentre project were randomly selected from population registers or electoral lists and stratified by sex and five year age groups. The planned sample size was 10,000 persons in each country. At baseline, a cross-sectional analysis of the random age- and sex-stratified population sample of males and females aged 45–69 years old was performed in 2003–2005 in Novosibirsk (Russia) (*n* = 9360, response rate 61%). In the Russian arm of the study, both the questionnaire and the examination have been completed in an outpatient clinic. The details of sampling have been described elsewhere [17]. The cohort was re-examined in 2006–2008 and in 2015–2017. Follow-up data were collected between 2003 and 2017; the average follow-up period comprised 13.7 (0.7) years (mean (SD)). The last survey and follow-up were supported by Russian Science Foundation. We excluded from the analysis those who have no baseline glucose assessment and those with T2DM at baseline defined as fasting plasma glucose (FPG) ≥7.0 mmol/L or current treatment with insulin or oral hypoglycaemic agents (1621 subjects excluded). In total, the final analysis included 7739 participants (3376 males; 4363 females). Incident DM in any wave of follow-up was registered as the endpoint.

### 2.2. Baseline Examination

The baseline and repeated examinations involved standardised questionnaires, objective measurements, and blood sampling for biomarker assays. Details of the baseline protocol of the HAPIEE project have been published previously [17,18]. The participants did an interview with trained technicians regarding sociodemographic characteristics, behavioural risk factors (including smoking and alcohol intake), a history of DM and hypertension and their treatment, a history of major CVDs and other chronic diseases, physical activity (PA), a family history of T2DM and CVDs, meal frequency and other health, lifestyle, and social characteristics.

Objective measurements included anthropometry (body weight, height, WC, and hip circumference) and blood pressure (BP) determination. The body weight was measured on a weighing scale with a participant wearing one layer of clothes (accuracy to 0.1 kg). The height was measured using a vertical stadiometer (accuracy 0.1 cm). WC and hip circumference were measured by means of a tape with 0.1 cm accuracy. The body–mass index (BMI) was calculated as weight in kilograms divided by the square of height in meters and categorised as BMI < 25.0 kg/m^2^ and BMI ≥ 25 kg/m^2^ [18]. Abdominal obesity was defined as a WC of ≥94 cm for males and ≥80 cm for females [3].

BP was measured after 5-min rest on the right hand in a sitting position using Omron M5-I (Omron Co. Ltd., Terado-cho, Muko, Kyoto, Japan). The measurement of BP was carried out three times with an interval of 2 min. For the present analysis, the average of the three values of BP was used. Hypertension was defined as systolic BP (SBP) ≥140 mmHg or diastolic BP (DBP) ≥90 mmHg and/or antihypertensive medication use during the last two weeks.

Blood samples were drawn after overnight fasting (at least 8 h). Serum levels of glucose, total cholesterol (TC), triglycerides (TGs), and high-density lipoprotein cholesterol (HDL-C) were determined by enzymatic methods on a KoneLab 30i automated analyser (Thermo Fisher Scientific Inc., Waltham, MA, USA). The fasting serum glucose value was converted to fasting plasma glucose (FPG) via the formula of the European Association for the Study of Diabetes in 2007 [19]:

FPG (mmol/L) = −0.137 + 1.047 × serum glucose concentration (mmol/L). Impaired fasting glucose was defined as FPG of 6.1–6.9 mmol/L.

Hypertriglyceridemia was defined as a serum TG level of ≥2.8 mmol/L, and abnormal HDL-C was defined as HDL cholesterol of ≤0.9 mmol/L for males and females [3].

We followed the cohort from the baseline survey up to 31 December 2017; the average period of follow-up comprised 13.7 (0.7) years (mean (SD)). The incident cases of T2DM were ascertained using overlapping sources—the cases registered by the municipal diabetes register and new cases identified by the repeated surveys in 2006–2008 and 2015–2017. Baseline T2DM was defined as FPG of ≥7.0 mmol/L or current treatment with insulin or oral hypoglycaemic agents [20]; these persons were excluded from the study. An incident case of DM was defined as a new case registered by the T2DM register during the 2003–2017 period or a new case identified in the second or third survey as FPG ≥7.0 mmol/L or current treatment with insulin or oral hypoglycaemic agents.

### 2.3. Statistical Analyses

These analyses were carried out using the statistical package SPSS for Windows Version 13.0, (SPSS Inc., Chicago, IL, USA). Baseline characteristics of the study participants are given as means (SD) and were compared by the χ^2^ test, unpaired Student’s *t* test (2-tailed), or Mann–Whitney test, depending on the type of distribution of the variables.

First, the association between potential risk factors and incident T2DM was assessed by Cox regression analysis in age- and sex-adjusted Model 1 and in age-adjusted Model 2 split by sex. Incident T2DM served as a dependent variable. Independent variables tested sequentially included the education level (categorised into three groups—higher education, secondary, or primary education); marital status (categorised into two groups—married or cohabitating/single); SBP and DBP (as continuous variables); hypertension (dichotomised as yes/no); TC, TG, HDL-C, and FPG (as continuous variables); high TG concentration (dichotomised as ≥2.8 mmol/L and <2.8 mmol/L); low HDL-C concentration (dichotomised as cholesterol ≤0.9 mmol/L for males and females and cholesterol >0.9 mmol/L for males and females); fasting hyperglycaemia (dichotomised as ≥6.1 and <6.1 mmol/L); obesity (categorised into two groups—BMI < 25 kg/m^2^ and ≥25 kg/m^2^); abdominal obesity (categorised into two groups—WC ≥ 94 cm for males and ≥80 cm for females and <94 cm for males and <80 cm for females); smoking (categorised into three groups—current smokers, past smokers, or never smoked); alcohol consumption was rated in two versions—as a continuous variable (the mean dose of ethanol per occasion, in grams) and as a dichotomised variable (higher than a calculated sex-specific mean amount of alcohol intake per session in the population sample, yes/no); the lack of leisure time PA weekly or daily (PA in a previous week was categorised into three groups—none, insufficient (1–179 min), and sufficient (≥180 min); everyday PA was categorised into three groups—none, insufficient (1–29 min), and sufficient (≥30 min)), and a dichotomised variable ‘low PA’ was generated based on weekly or daily insufficient PA at leisure time (yes/no); fruit and vegetable consumption (dichotomised as every day/not every day); a family history of DM (dichotomised as a T2DM history in first-degree relatives and no family history of T2DM); and a history of major CVDs (dichotomised as yes/no). Covariates were age (as a continuous variable, per year) and sex (male or female).

Among the tested factors, for subsequent Cox regression analysis, we selected those significantly associated with T2DM, and in a set of similar variables, we selected the ones with greater hazard ratios (HRs). The selected variables included age, BMI, abdominal obesity, hypertension, dyslipidaemia (high TG and low HDL-C levels), FPG, the history of CVDs, smoking status, alcohol intake, the education level, marital status, PA, fruit and vegetable consumption, and the family history of DM.

At the second stage, we applied Cox proportional hazards regression analysis to assess the association between risk factors and incident T2DM in age-adjusted and multivariable-adjusted models separately in males and females.

HRs with a 95% confidence interval (CI) were calculated for the above factors selected as independent variables. In males, the multivariable model included age, BMI ≥ 25 kg/m^2^, hypertension, high TG concentration, FPG, a family history of DM, and a history of CVD. In females, the model included age, BMI ≥ 25 kg/m^2^, hypertension, high TG concentration, FPG, education level, PA, marital status, a family history of DM, and a history of CVD.

At the third stage, we used dichotomised variables based on cut-off points for risk factors of T2DM obtained using receiver-operating characteristic (ROC) analysis. These cut-offs were applied further to build a 10-year risk score for T2DM using Cox regression and ROC analysis and select a model which includes a minimum number of prognostic parameters and has the maximum positive predictive power for T2DM risk.

Logistic regression was used to compute β-coefficients for significant risk factors for T2DM. Coefficients (β) of the model were used to assign a score value for each variable, and the composite diabetes risk score was calculated as the sum of those scores. The sensitivity (the probability that the test is positive for subjects who will receive drug-treated diabetes in the future) and the specificity (the probability that the test is negative for subjects without drug-treated diabetes) with 95% CIs were calculated for each diabetes risk score level in differentiating subjects developed incident T2DM from those who did not. Then, ROC curves were plotted for the diabetes risk score; the sensitivity was plotted on the *y*-axis, and the false-positive rate (1-specificity) was plotted on the *x*-axis. The more accurately discriminatory the test, the steeper the upward portion of the ROC curve and the higher the area under the curve (AUC), the optimal cut point being the peak of the curve.

## 3. Results

The population sample of males and females aged 45–69 years old was examined at baseline in 2003–2005 in Novosibirsk (*n* = 9360 subjects). The present analysis was limited to those without T2DM at baseline (*n* = 7739). The baseline characteristics of this sample are presented in Table 1.

### 3.1. The 14-Year Risk of T2DM

During the follow-up of 13.7 (0.7) years (mean (SD)), 915 participants developed T2DM for the first time (11.8%). Among males, the frequency of incident cases of T2DM was 1.8-fold lower than that among females, 9.7% and 15.5%, respectively (*p* < 0.0001).

We compared 26 factors potentially related to T2DM between the participants who developed T2DM and those who remained free of T2DM; the results are summarised in Table 2. Individuals of both sexes who developed T2DM were younger, had a greater BMI, greater WC, more frequent abdominal obesity, higher SBP and DBP, more frequent hypertension, a more frequent family history of DM, and higher FPG, TG, and HDL-C levels as compared to those without T2DM.

Males with incident T2DM had higher TC levels were less frequently current smokers and more frequently smokers in the past than their counterparts without T2DM. Females with incident T2DM had a more frequent history of CVD, engaged in less PA, and more frequently had a secondary-education level than their counterparts without T2DM.

The results of the Cox regression analysis are presented in Table 3. In the age-adjusted model for males, the 14-year risk of incident T2DM was associated with BMI ≥ 25 kg/m^2^, abdominal obesity, fasting hyperglycaemia, a high TG level, hypertension, and a family history of DM (Table 3). In the age-adjusted model for females, the 14-year risk of incident T2DM was associated with abdominal obesity, BMI ≥ 25 kg/m^2^, fasting hyperglycaemia, a high TG level, hypertension, a family history of DM, any secondary -education level, low PA (1–179 min/week or 1–29 min/day), and a history of CVD (Table 3).

The results of multivariable-Cox regression analysis are given in Table 3. In the multivariable-adjusted model, BMI ≥ 25 kg/m^2^ made the greatest contribution to the development of T2DM, with HR = 3.34 (2.60; 4.30); besides, the risk of incident T2DM was independently associated with fasting hyperglycaemia (HR = 2.70 (2.35; 3,10)), hypertension (HR = 1.86 (1.57; 2.21)), a high TG level (HR = 1.59 (1.26; 2.01)), a family history of DM (HR = 1.53 (1.28; 1.83)), a low leisure-time PA (HR = 1.22 (1.02; 1.46)) (Table 3).

### 3.2. Development of the Type 2 Diabetes Risk Scale

In many countries, 10-year risk scales for T2DM have been created on the basis of epidemiological studies [11]. These scales are based on the most specific risk factors of T2DM in a population under study. To build models for assessing the risk of T2DM, we used cut-off points (cut-off) which were calculated for the Siberian population in the age group under study. This allowed us to take into account the regional characteristics of the studied cohort. Different values were obtained for males and females: for males, the BMI (cut-off) was 27 kg/m^2^, WC (cut-off) 95.0 cm, SBP (cut-off) 150 mmHg, DBP (cut-off) 90 mmHg, TG (cut-off) 1.4 mmol/L, HDL-C (cut-off) 0.9 mmol/L, and FPG (cut-off) 6.0 mmol/L; for females, the BMI (cut-off) 32 kg/m^2^, WC (cut-off) 95 cm, SBP (cut-off) 135 mmHg, DBP (cut-off) 90 mmHg, TG (cut-off) 1.5 mmol/L, HDL-C (cut-off) 0.9 mmol/L, and FPG (cut-off) 5.7 mmol/L.

We designed a model for assessing the 10-year risk of T2DM on the basis of the cut-offs and multivariate Cox regression analysis. To create a risk scale, a new variable was created—arterial hypertension (HT)_1_ BP ≥ 150/90 mmHg for males and BP ≥ 135/90 mmHg for females. During the 10 years of follow-up, 463 (5%) new cases of T2DM were registered. The average age at first diagnosis of T2DM in the study population was 61.3 (6.7) years old.

Having studied the association between dichotomised risk factors and the 10-year risk of T2DM by multivariable-adjusted Cox regression analysis.

In males, the final version of the T2DM risk model includes significant risk factors dichotomised by the cut-off as T2DM predictors: FPG (cut-off) ≥6.0 mmol/L (HR = 3.79 (2.6; 5.6)), the BMI (cut-off) ≥27 kg/m^2^ (HR = 3.03 (2.0; 4.7)), HDL-C (cut-off) ≤0.9 mmol/L (HR = 2.20 (1.2; 3.9)), TG (cut-off) ≥1.4 mmol/L (HR = 1.55 (1.0; 2.3)), and HT_1_ ≥150/90 mmHg (HR = 1.57 (1.0; 2.4). The model for males is adjusted for FPG (cut-off), BMI (cut-off), HDL-C (cut-off), TG (cut-off), HT_1._ For females, predictors were included that were different from those in the model for males: WC (cut-off) ≥95 cm (HR = 2.25 (1.6; 3.1)), FPG (cut-off) ≥5.7 mmol/L (HR = 2.58 (2.0; 3.3)), TG (cut-off) ≥1.5 mmol/L (HR = 1.81 (1.4; 2.3)), HT_1_ ≥135/90 mmHg (HR = 1.64 (1.2; 2.2)), a family history of T2DM (HR = 1.50 (1.1; 2.0)), and the BMI (cut-off) ≥32 kg/m^2^ (HR = 1.47 (1.1; 1.9)). The model for females is adjusted for WC (cut-off), FPG (cut-off), TG (cut-off), HT_1_, age, family history of T2DM, BMI (cut-off).

Exp(B) measures served as weights to create the risk scale. Each predictor included in the regression model was scored by rounding out Exp(B) to a whole number (Table 4). The maximum total number of points on the created T2DM risk scale of the model is 13 for males and 12 for females.

To determine the threshold of the total score associated with a high risk of T2DM, a receiver-operating characteristic (ROC) curve was constructed. The optimal cut-off for the sum of points that allowed to divide the analysed groups into two subgroups was 7 points (sensitivity (Se) 76.0%, specificity (Sp) 71.5%) for males and 6 points (Se 71.7%, Sp 69.2%) for females. When cross-checking the adequacy of the model in the population of Novosibirsk, we calculated the actual incidence of T2DM. Among the males who scored 7 or more points, T2DM developed in 10.2% of cases, and in the group of people who scored less than 7 points, only in 1.4% of cases; females who scored 6 or more points developed T2DM in 15.8% of cases, and among those who scored less than 6 points, incident cases of DM were detected only in 3.2% of cases.

Clinical testing of the newly developed model and of the Finnish model predicting the 10-year risk of T2DM on persons of retirement age in Novosibirsk revealed difficulties with independent filling out of the questionnaire [21]. Determination of the BMI, blood lipid levels, and in some cases, of own BP was difficult for the elderly. Accordingly, the next aim was to develop a simple T2DM risk calculator that would be convenient to use in primary health care and for self-completion for both sexes. Our model had to include only the parameters that can be easily assessed without laboratory tests or other measurements that require specialised medical skills. Predictors of the 10-year risk of T2DM selected for the best scale included history of hypertension 1.6 (HR = 1.6 (1.3; 1.8)), family history of T2DM (HR = 1.8 (1.1; 1.9)), WC (cut-off) (HR = 3.6 (1.9; 3.8)).

As a result of the analysis of various multivariate models, a scale with the best quality was selected (Table 5), where the area under the curve (AUC) was 0.71; this scale included such risk factors as WC, a history of arterial hypertension, and a family history of T2DM.

The relative risk scores obtained in the Cox regression analysis were chosen as variables to create a risk scale. Each of the three predictors included in the regression model was scored by rounding out to a whole number (Table 5). The highest total score on the created T2DM risk scale is 8 points. The cut-off of the scale was found to be 4 points—Se 74.7% and Sp 60.0%.

In the group that scored ≥4 points during 10 years, T2DM developed in 10.7% of cases, and in the group with <4 points, T2DM developed in 2.6% of cases.

## 4. Discussion

According to our analysis of the study population aged 45–69 years old, the prevalence of T2DM was 11.0% in both the male and female samples [22]. During 14 years of observation, new cases of T2DM occurred in 9% of the population more often among females than among males, 328 (9.7%) and 587 (15.5%), respectively (*p* < 0.001).

The results were subjected to Cox univariate proportional hazards regression analysis with adjustment for age. This analysis revealed significant risk factors for T2DM among males and females: BMI ≥ 25 kg/m^2^, abdominal obesity, fasting hyperglycaemia, a high TG level, hypertension, a family history of DM, and a history of CVD. Among females, additional predictors were vocational- or primary-education level and leisure-time PA reported for the previous week (insufficient: 1–179 min per week or 1–29 min every day).

An increased BMI and WC indicate the presence of increased intra-abdominal visceral fat, which disrupts insulin metabolism through a release of serum-free fatty acids [23]. Nonetheless, according to a meta-analysis in 2018, not all obese individuals are at the same risk of T2DM; it seems that the risk is affected by their metabolic profile—the metabolically unhealthy obese have a ~10-fold higher risk of T2DM, whereas the metabolically healthy obese have a ~4.5-fold higher risk of T2DM, as compared to nonobese individuals. Moreover, in that study, weight gain during early adulthood was found to be more harmful than weight gain after the age of 25. On the contrary, peripheral fat accumulation has been linked to a better metabolic profile, which manifests itself in the observed protective effect of the greater hip circumference on T2DM [24].

Hypertension is known to be associated with the development of T2DM [25,26]. Persons with hypertension have an increased activity of the renin–angiotensin system, which causes systemic inflammatory processes leading to T2DM [27].

The presence of a family history of DM indicates a genetic contributor to DM but can also reflect the lifestyle or environmental conditions people were exposed to during their upbringing [28].

Impaired lipid metabolism with insulin resistance is characterised by an increase in TG levels, lower concentration of HDL-C, and an increase in free fatty acid levels. Diabetic dyslipidaemia also includes qualitative and kinetic lipid disorders, which are more atherogenic in nature, because cholesterol ester transfer protein (CETP) increases the production of small particles of low-density lipoproteins (LDL) [29]. Earlier studies in mice have shown dyslipidaemia to be a factor contributing to the apoptosis of pancreatic β-cells, to insulin biosynthesis, defective insulin secretion, and altered glucose metabolism. Fatty acid metabolism is known to be affected by ceramide formation, endoplasmic reticulum stress, oxidative stress, inflammation, the insulin signalling pathway, and protein kinase B, associated with damage to pancreatic β-cells. According to observational studies, there is a correlation between the level of TGs and the risk of T2DM [30,31].

High PA at leisure time reduces the relative risk of T2DM. Regular PA improves blood glucose control and can prevent or delay the onset of T2DM. [32,33]. Observational studies suggest that greater physical performance is associated with a reduced risk of T2DM [23], even if only as moderate-intensity exercises.

A 2018 meta-analysis revealed an association of lower educational attainment with a higher risk of T2DM [34]. The education level constitutes a component of socioeconomic status. Lower socioeconomic status correlates with higher stress levels, leading to a disruption of endocrine function through perturbations in the neuroendocrine system. Additionally, people with low socioeconomic status are more prone to an unhealthy lifestyle and have limited access to healthcare facilities [35].

In a multivariate regression analysis, we identified gender differences in the risk factors of T2DM. Among males, the best predictors were BMI, fasting hyperglycaemia, hypertension, and a family history of DM. Among females, the best predictors were BMI, fasting hyperglycaemia, hypertension, and fasting hyperglycaemia.

In 2016, Kautzky-Willer et al. analysed sex dimorphism in diabetes risk factors and found that T2DM is more often diagnosed at an earlier age in males, whereas the best predictors of T2DM are the BMI and WC in males and the BMI and WC in females. Limited mobility increases the risk of T2DM in females, and fasting hyperglycaemia in males [36]. The obtained data are explained by those authors as the influence of sex hormones on energy metabolism, body composition, vascular function, and inflammatory reactions. An endocrine imbalance denotes adverse cardiometabolic symptoms in females or males. Furthermore, genetic effects, epigenetic mechanisms, nutritional factors, and a sedentary lifestyle have different effects on the risk of T2DM and complications in both sexes [36].

According to the literature, the risk of DM increases with age [37], although in our models no such increase in risk was found. A possible explanation is that other factors such as WC or physical inactivity also increase with age [38], thus cancelling out the age-specific increase in DM risk.

According to a prospective study in Turkey with an average follow-up of 5.9 years, significant independent predictors of DM are abdominal obesity (risk ratio = 2.61 (95% CI 1.87–3.63)) and age in both sexes, hypertension (risk ratio = 1.81 (95% CI 1.10–2.98)), and low HDL-C in males only [39].

In the FINDRISK study, 38,689 participants aged 30–59 years old were analysed to estimate the prevalence of T2DM at the start and within 10 years. Among males, the frequency of diagnosed pharmacologically controlled T2DM increased over time. Compared to males surveyed in the 1970s, the incidence of diabetes was higher among males in the 1980s (adjusted HR = 1.44, 95% CI: 1.13–1.84) and in the 1990s (adjusted HR = 1.72, 95% CI: 1.32–2.24). The BMI explained some but not all of this variance. The increase occurred predominantly among males with a low education level (adjusted HR in the 1980s = 2.07, 95% CI: 1.28–3.35; adjusted HR in the 1990s = 2.12, 95% CI: 1.28–3.53) and an average education level (adjusted HR in the 1980s = 1.30, 95% CI: 0.85–1.99; adjusted HR in the 1990s = 1.65, 95% CI: 1.05–2.60). The female subgroup showed no dependence of DM incidence on education [40].

In a study conducted in Iran, after observation for an average of six years, 237 new cases of diabetes were identified, which corresponded to an age- and sex-adjusted cumulative incidence of 6.4% (95% CI: 5.6–7.2). In that study, in addition to the classic risk factors of DM, female sex and low levels of education significantly increased the risk of DM in the age-adjusted models. In the full model, independent predictors were age (odds ratio = 1.2 (95% CI: 1.1–1.3), family history of DM (1.8 (1.3–2.5)), BMI ≥ 30 kg/m^2^ (2.3 (1.5–3.6)), abdominal obesity (1.9 (1.4–2.6)), high TG concentration (1.4 (1.1–1.9)), impaired fasting glucose (7.4 (3.6–15.0), impaired glucose tolerance (5.9 (4.2–8.4)), and combined impaired fasting glucose and impaired glucose tolerance (42.2 (23.8–74.9)) [41].

In Australia, among 554 adults who completed the study with a six-year follow-up, 100 developed DM. Abdominal obesity increased the risk of DM for aboriginal people (risk ratio = 2.0 (95% CI: 1.1–3.6)) and for residents of the Torres Strait Islands (odds ratio = 6.3 (95% CI: 2.5–16.1)) as compared to subjects with normal weight. Metabolic syndrome was a strong predictor of DM (corrected risk ratio = 2.4 (CI 95% 1.6–3.7)). For both groups, the ratio of WC to hip circumference and the presence of metabolic syndrome predicted DM better than did WC or the BMI [42].

Risk models and calculators were first developed for cardiovascular diseases and are widely used in clinical practice and public health.

Diabetes risk scales in various countries have been actively designed since the 2000s. Most risk scales are based on the most sensitive and easily identifiable risk factors of T2DM such as age, sex, BMI, WC, T2DM in relatives, PA level, hypertension or regular use of antihypertensive drugs, and occasionally detectable hyperglycaemia. According to a 2011 review, 145 T2DM risk scales had been published, of which 94 risk models were consistent with qualitative studies—overall, 55 were inferences from risk models based on population studies and 39 were based on validation in new populations [10]. Millions of participants worldwide have already taken part in epidemiological studies assessing the risk of DM. A greater number of possible risk scales are now available to those wishing to use them clinically, but none of them is perfect, all have strengths and weaknesses.

The study has several limitations. One potential limitation is related to non-responders at baseline who might differ from responders by the distribution of DM2 risk factors or poor health; but, the response rate (61%) was a commonly accepted level for population studies and it is unlikely to influence the estimates of outcome risk. Against potential ‘recall’ bias in the survey, we applied standardised questionnaires/personnel/interview procedures, duplicated questions, and definitions of diseases by few sources (e.g., T2DM by interview of DM history and treatment, and fasting plasma glucose). We could not exclude the attrition bias at the follow-up stage due to those who dropped-out from the follow-up as non-responders for waves 2 or 3 or missed from the DM register, and those who died from competing causes. However, we used overlapping sources for diabetic endpoints in the cohort (DM register, CVD and mortality register, two repeated surveys, phone calls for non-responders) which minimised the chance for missing new cases of T2DM. The study was conducted in an urban Siberian population and has limited generalisability. However, it fits well for the risk of T2DM in the region and provides an excellent approach from the point of personalised medicine for region-specific preventive measures.

The validation of the FINDRISC questionnaire in Novosibirsk demonstrated the good quality of the model, which provides support for its use in Siberian populations. The cut-off risk score of 11 using the FINDRISC questionnaire to identify diabetes had a sensitivity and specificity (76.0% and 60.2%, respectively). The area under the receiver-operating curve for T2DM was 0.73 (0.73 in men and 0.70 in women) [43]. The T2DM risk model that includes three risk factors has several advantages over existing models. The cut-off of the scale was found to be 4 points—Se 74.7% and Sp 60.0%. Thus, the indicators of sensitivity and specificity of the cut-off values of the FINDRISK risk scale and the short Siberian risk scale are comparable and can be used in the medical practice.

## 5. Conclusions

Millions of people around the world have already participated in epidemiological studies aimed at assessing the risk of DM. A large number of risk scales are now available to those who seek to apply them clinically, but none of these instruments is ideal; each has strengths and shortcomings. In 2015, validation of the FINDRISC risk scale was conducted on a population sample in Novosibirsk [43]. During the validation, we obtained data suggestive of good quality of the model, which allows us to recommend its use in the Siberian population. Nevertheless, according to our data, not all risk factors included in the FINDRISC scale are widespread among patients with newly diagnosed T2DM, and the incidence of newly diagnosed DM in the very-high-risk group is lower than that predicted in Finland (22.6% versus 50%) [43]. Thus, the task of creating a risk scale for DM remains urgent, as does the issue of finding risk factors with a pronounced contribution to the development of T2DM in the Siberian population representing the appropriate approach from the point of personalised medicine. In addition, the revealed risk determinants might be validated on a wider sample for generalisability. In this study, for the first time in Russia, as part of a cohort study, an assessment of the risk factors of T2DM in males and females was carried out via multivariate risk models for the development of T2DM. This approach allowed us to identify significant risk factors and direct preventive measures for correcting these factors.

The T2DM risk model that includes three risk factors has several advantages over existing models. It is based on a questionnaire, which takes little time to complete and enables a person to independently determine their risk of T2DM and then visit a healthcare institution for examination, determination of carbohydrate metabolism disorders or T2DM, and preventive measures. 

## 6. Patents

Patent No. 030585 (EAPO), issued on 31.08.2018. “A method for predicting the risk of developing type 2 diabetes”.

## Figures and Tables

**Table 1:** Characteristics of the studied population sample (aged 45–69 years old at baseline, *n* = 7739).

	Males and Females	Males	Females	*p*
Examined	7739	3376	4363	
Age (years)	57.7 (7.1)	57.8 (7.0)	57.6 (7.1)	0.211
Height (cm)	164.0 (8.9)	171.3 (6.3)	158.4 (6.0)	<0.001
Weight (kg)	75.8 (14.2)	77.3 (13.8)	74.7 (14.4)	<0.001
BMI (kg/m^2^)	28.3 (5.3)	26.3 (4.2)	29.8 (5.6)	<0.001
Waist circumference (cm)	91.7 (12.4)	93.0 (11.6)	90.7 (12.9)	<0.001
Abdominal obesity ^▪^, *n*(%)	5014 (64.8)	1563 (46.3)	3451 (79.1)	<0.001
SBP (mmHg)	142.8 (24.5)	142.4 (22.6)	143.1 (25.8)	0.202
DBP (mmHg)	90.0 (13.3)	90.0 (13.0)	89.9 (13.5)	0.899
FPG (mmol/L)	5.61 (0.6)	5.63 (0.6)	5.59 (0.6)	0.003
TC (mmol/L)	6.2 (1.2)	5.9 (1.1)	6.4 (1.2)	<0.001
TG (mmol/L)	1.4 (0.7)	1.4 (0.7)	1.5 (0.6)	<0.001
HDL-C (mmol/L)	1.5 (0.4)	1.5 (0.4)	1.6 (0.3)	<0.001
Hypertension * *n*(%)	4910 (63.5)	2082 (61.7)	2828 (64.8)	0.005
Low HDL-C ^■^, *n*(%)	43 (0.6)	26 (0.8)	17 (0.4)	0.026
High TG ^♦^, *n*(%)	296 (3.8)	125 (3.7)	171 (3.9)	0.609
Fasting hyperglycaemia ^●^, *n*(%)	1541 (20.2)	708 (21.2)	833 (19.4)	0.045
BMI ≥ 25 kg/m^2^, *n*(%)	5510 (71.2)	1993 (59.0)	3517 (80.6)	<0.001
Alcohol, mean dose per occasion (g)	35.8 (36.5)	54.2 (45.6)	21.7 (17.3)	<0.001
**Smoking, *n*(%)**				<0.001
Never smoked	4624 (59.8)	917 (27.2)	3707 (85.0)	
Former smoker	973 (12.6)	786 (23.3)	187 (4.3)	
Current smoker	2141 (27.7)	1672 (49.5)	469 (10.7)	
**Marital status, *n*(%)**				<0.001
Single	2145 (27.7)	387 (11.5)	1758 (40.3)	
Married or cohabitating	5594 (72.3)	2989 (88.5)	2605 (59.7)	
**Education level, *n*(%)**				<0.001
Only primary	719 (9.3)	335 (9.9)	384 (8.8)	
Any secondary	4722 (61.0)	1927 (57.1)	2795 (64.1)	
University	2298 (29.7)	1114 (33.0)	1184 (27.1)	
Family history of T2DM (%)	863 (11.2)	300 (9.0)	563 (12.9)	<0.001
History of CVD, *n*(%)	832 (10.8)	475 (14.1)	357 (8.2)	<0.001
Fruit and vegetable consumption less than every day ^, *n*(%)	833 (10.8)	361 (10.7)	472 (10.8)	0.856
Leisure-time PA in previous week ^#^, min	6260 (80.9)	2803 (83.0)	3457 (79.2)	<0.001

^▪^ Abdominal obesity: waist circumference (WC) ≥94 cm for males and ≥80 cm for females. * Hypertension is defined as systolic blood pressure (SBP) ≥140 mmHg or diastolic blood pressure (DBP) ≥90 mmHg or antihypertensive drug treatment during the last two weeks; ^■^ Low high-density lipoprotein cholesterol (HDL-C) ≤0.9 mmol/L for males and females. ^♦^ High triglyceride (TG): serum TG concentration ≥2.8 mmol/L. ^●^ Fasting hyperglycaemia: glucose ≥6.1 mmol/L. ^ Fruit and vegetable consumption (every day/not every day); ^#^ Leisure time physical activity (PA) in a previous week (insufficient: 1–179 min or sufficient: ≥180 min).

**Table 2:** Baseline characteristics of groups with incident T2DM and without T2DM stratified by sex (mean (SD), and *n*(%)).

	Males		*p*	Females		*p*
	T2DM (+)	T2DM (−)		T2DM (+)	T2DM (−)	
**Examined**	328	3048		587	3776	
Age (years)	57.2 (6.7)	57.8 (7.1)	0.154	57.3 (6.7)	57.6 (7.2)	0.347
Height (cm)	171.5 (6.4)	171.3 (6.3)	0.572	158.2 (5.7)	158.4 (6.1)	0.595
Weight (kg)	87.1 (14.5)	76.2 (13.3)	<0.001	83.0 (14.7)	73.4 (14.0)	<0.001
BMI (kg/m^2^)	29.6 (4.2)	25.9 (4.0)	<0.001	33.1 (5.5)	29.3 (5.4)	<0.001
Waist circumference (cm)	101.9 (11.1)	92.1 (11.2)	<0.001	98.9 (11.8)	89.4 (12.6)	<0.001
Abdominal obesity ▪, *n*(%)	255 (78.0)	1308 (42.9)	<0.001	556 (94.7)	2895 (76.7)	<0.001
SBP (mmHg)	146.9 (22.8)	142.0 (22.5)	<0.001	149.7 (25.6)	142.1 (25.7)	<0.001
DBP (mmHg)	93.9 (13.1)	89.6 (13.0)	<0.001	94.1 (13.0)	89.3 (13.5)	<0.001
FPG (mmol/L)	6.0 (0.6)	5.6 (0.6)	<0.001	5.9 (0.6)	5.5 (0.6)	<0.001
TC (mmol/L)	6.1 (1.1)	5.9 (1.1)	0.003	6.5 (1.2)	6.4 (1.2)	0.181
TG (mmol/L)	1.7 (0.9)	1.3 (0.6)	<0.001	1.8 (0.9)	1.4 (0.6)	<0.001
HDL-C (mmol/L)	1.4 (0.3)	1.5 (0.4)	<0.001	1.5 (0.3)	1.6 (0.3)	<0.001
Hypertension *, *n*(%)	248 (75.6)	1834 (60.2)	<0.001	479 (81.6)	2349 (62.2)	<0.001
Low HDL-C ^■^, *n*(%)	3 (0.9)	23 (0.8)	0.754	4 (0.7)	13 (0.3)	0.216
High TG ^♦^, *n*(%)	29 (8.8)	96 (3.2)	<0.001	62 (10.7)	109 (2.9)	<0.001
Fasting hyperglycaemia ^●^, *n*(%)	149 (47.2)	559 (18.5)	<0.001	247 (43.5)	586 (15.7)	<0.001
BMI ≥ 25 kg/m^2^, *n*(%)	287 (87.5)	1706 (56.0)	<0.001	556 (94.7)	2961 (78.4)	<0.001
Alcohol, mean dose per occasion (g)	54.3 (46.1)	54.2 (45.5)	0.953	22.3 (19.2)	21.6 (17.0)	0.322
**Smoking, *n*(%)**			0.001			0.423
Never smoked	101 (30.9)	816 (26.8)		509 (86.7)	3198 (84.7)	
Former smoker	96 (29.4)	690 (22.6)		21 (3.6)	166 (4.4)	
Current smoker	130 (39.8)	1542 (50.6)		57 (9.7)	412 (10.9)	
**Marital status, *n*(%)**			0.229			0.294
Single	31 (9.5)	356 (11.7)		230 (39.2)	1528 (40.5)	
Married or cohabitating	297 (90.5)	2692 (88.3)		357 (60.8)	2248 (59.5)	
**Education level, *n*(%)**			0.287			0.001
Primary	26 (7.9)	309 (10.1)		43 (7.3)	341 (9.0)	
Any secondary	184 (56.1)	1743 (57.2)		417 (71.0)	2378 (63.0)	
University	118 (36.0)	996 (32.7)		127 (21.6)	1057 (28.0)	
Family history of T2DM (%)	44 (13.5)	256 (8.5)	0.002	117 (20.0)	446 (11.9)	<0.001
History of CVD, *n*(%)	52 (15.9)	423 (13.9)	0.328	61 (10.4)	296 (7.8)	0.036
Fruit and vegetable consumption less than every day ^, *n*(%)	35 (10.7)	326 (10.7)	0.993	66 (11.2)	406 (10.8)	0.740
Leisure-time PA in previous week ^#^, min	276 (84.1)	2527 (82.9)	0.570	490 (83.5)	2967 (78.6)	0.006

^▪^ Abdominal obesity: WC ≥ 94 cm for males and ≥80 cm for females. * Hypertension is defined as SBP ≥ 140 mmHg or DBP ≥ 90 mmHg or antihypertensive drug treatment during the last 2 weeks; ^■^ Low HDL-C: ≤0.9 mmol/L for males and females. ^♦^ High TG: serum TG level ≥2.8 mmol/L. ^●^ Fasting hyperglycaemia: glucose concentration ≥6.1 mmol/L. ^ Fruit and vegetable consumption (every day/not every day); ^#^ Leisure time PA in a previous week (insufficient: 1–179 min or sufficient: ≥180 min).

**Table 3:** The preliminary assessment of the relationship between the 14-year risk of T2DM and risk factors by age-adjusted and age- and sex-adjusted Cox regression.

Risk Factors	Model 1 HR (95% CI) *	Model 2		Model 3
		HR (95% CI) **	HR (95% CI)	HR (95% CI) ***
	Males and Females	Males	Females	Males and Females
**Smoking**				
Never	1.0	1.0	1.0	1.0
Former	1.02 (0.81; 1.27)	1.10 (0.83; 1.45)	0.74 (0.48; 1.15)	0.84 (0.67; 1.06)
Current	0.72 (0.60; 0.88)	0.66 (0.50; 0.85)	0.87 (0.66; 1.15)	0.83 (0.68; 1.02)
**Alcohol intake above sex-specific mean amount per session in study population**				
Alcohol, <mean dose per occasion (g)	1.0	1.0	1.0	
Alcohol, ≥mean dose per occasion (g)	0.99 (0.87; 1.13)	0.93 (0.74; 1.16)	1.02 (0.87; 1.21)	
**Education level**				
University	1.0	1.0	1.0	1.0
Any secondary	1.21 (1.04; 1.40)	0.91 (0.72; 1.15)	1.44 (1.18; 1.76)	1.05 (0.79; 1.38)
Primary	0.95 (0.73; 1.25)	0.80 (0.52; 1.22)	1.10 (0.77; 1.57)	1.25 (0.96; 1.62)
**Marital status**				
Single	1.0	1.0	1.0	
Married or cohabitating	1.07 (0.92; 1.25)	1.21 (0.83; 1.75)	1.05 (0.89; 1.24)	
**Family history of DM**				
No	1.0	1.0	1.0	1.0
Yes	1.65 (1.34; 1.96)	1.57 (1.14; 2.16)	1.69 (1.38; 2.07)	1.53 (1.28; 1.83)
**History of CVD**				
No	1.0	1.0	1.0	1.0
Yes	1.31 (1.07; 1.61)	1.22 (0.90; 1.66)	1.40 (1.07; 1.84)	1.04 (0.85; 1.28)
Hypertension *				
No	1.0	1.0	1.0	1.0
Yes	2.56 (2.17; 3.01)	2.25 (1.74; 2.91)	2.82 (2.27; 3.49)	1.86 (1.57; 2.21)
**Abdominal obesity**				
No	1.0	1.0	1.0	
Yes	4.62 (3.74; 5.71)	4.38 (3.37; 5.69)	5.13 (3.56; 7.37)	
**BMI ≥ 25 kg/m^2^**				
No	1.0	1.0	1.0	1.0
Yes	4.87 (3.82; 6.21)	5.02 (3.62; 6.96)	4.70 (3.27; 6.75)	3.34 (2.60; 4.30)
**High TG level**				
No	1.0	1.0	1.0	1.0
Yes	2.93 (2.35; 3.65)	2.34 (1.58; 3.49)	3.27 (2.50; 4.26)	1.59 (1.26; 2.01)
**Low HDL-C concentration**				
No	1.0	1.0	1.0	1.0
Yes	1.49 (0.70; 3.15)	1.29 (0.41; 4.02)	1.68 (0.61; 4.64)	1.48 (0.70; 3.12)
**Fasting hyperglycaemia**				
No	1.0	1.0	1.0	1.0
Yes	3.51 (3.07; 4.02)	3.31 (2.65; 4.14)	3.61 (3.06; 4.27)	2.70 (2.35; 3.10)
**Low PA at leisure time**				
≥180 min per week	1.0	1.0	1.0	1.0
1–179 min per week	1.29 (1.08; 1.53)	1.11 (0.83; 1.50)	1.39 (1.12; 1.73)	1.22 (1.02; 1.46)
**Fruit and vegetable consumption**				
every day	1.0	1.0	1.0	1.0
not every day	0.97 (0.79; 1.19)	0.92 (0.65; 1.31)	0.99 (0.77; 1.28)	1.05 (0.85; 1.29)

* Model 1: age- and sex-adjusted model. ** Model 2: age-adjusted model split by sex. *** Model 3: The model is multivariable and adjusted for age, sex, a family history of diabetes mellitus (DM), fasting hyperglycaemia, a history of cardiovascular disease (CVD), hypertension, abdominal obesity, high TG level, low HDL-C concentration, education level, smoking, low PA, and fruit and vegetable consumption.

**Table 4:** The 10-year risk scale for incident T2DM.

T2DM Predictor		Interval Scale (Category)	Points
**Males**			
1	Fasting plasma glucose level >6.0 mmol/L	No	0
		Yes	4
2	BMI ≥ 27 kg/m^2^	No	0
		Yes	3
3	HDL-C level ≤0.9 mmol/L	No	0
		Yes	2
4	TG level ≥1.4 mmol/L	No	0
		Yes	2
5	BP level ≥150/90 mmHg	No	0
		Yes	2
**Females**			
1	WC ≥ 95 cm	No	0
		Yes	2
2	Fasting plasma glucose level ≥5.7 mmol/L	No	0
		Yes	3
3	TG level ≥ 1.5 mmol/L	No	0
		Yes	2
4	BP level ≥ 135/90 mmHg	No	0
		Yes	2
5	family history of T2DM	No	0
		Yes	2
6	BMI ≥ 32 kg/m^2^	No	0
		Yes	1

**Table 5:** The risk scale predicting the development of T2DM within 10 years (both females and males).

	T2DM Predictor	Interval Scale (Category)	Points
1	WC ≥ 95 cm	No	0
		Yes	4
2	Have you been told that you have high BP?	No	0
		Yes	2
3	Family history of T2DM	No	0
		Yes	2

Note: WC of 95 cm was obtained by receiver-operating characteristic (ROC) analysis for those surveyed who experienced the first onset of type 2 diabetes mellitus (T2DM) within 10 years of observation in Novosibirsk.

## Data Availability

The data presented in this study are available in tabulated form on request. The data are not publicly available due to ethical restrictions and project regulations.
